# Investigating the Predictive Performance of Process Data and Result Data in Complex Problem Solving Using the Conditional Gradient Boosting Algorithm

**DOI:** 10.3390/jintelligence13030029

**Published:** 2025-02-28

**Authors:** Fatma Nur Aydin, Kubra Atalay Kabasakal, Ismail Dilek

**Affiliations:** 1 Ministry of National Education, Ankara 06530, Türkiye; 2 Faculty of Education, Hacettepe University, Ankara 06530, Türkiye; katalay@hacettepe.edu.tr; 3 Galen College of Nursing, Lousville, KY 55129, USA; idilek@galencollege.edu

**Keywords:** process data, log files, result data, complex problem solving, conditional gradient boosting algorithm

## Abstract

This study aims to examine the predictive performance of process data and result data in complex problem-solving skills using the conditional gradient boosting algorithm. For this purpose, data from 915 participants of the 2012 cycle of the Programme for International Student Assessment (PISA) were utilized. Process data were obtained from the log file of the first question in the climate control unit task included in the problem-solving assessment of PISA 2012. Various cognitive and affective attributes from the same assessment were used as the result data. According to the results, (1) process data demonstrated a moderate, result data demonstrated a moderate-to-good, and process + result data demonstrated a good prediction performance. (2) The most effective variables were the VOTAT (vary-one-thing-at-a-time) strategy score and total time in process data; the mathematical literacy and reading literacy scores in result data; and the mathematical literacy and VOTAT strategy score in process + result data. The dominance of the mathematical literacy has been noteworthy.

## 1. Introduction

The primary purpose of measurement and evaluation practices is to validly and reliably demonstrate individuals’ competencies related to the latent trait targeted for measurement (Zhang et al. 2023). To this end, drawing conclusions is commonly based on the scores obtained from the items. The data obtained from the individuals’ item responses can be referred to as the result data (Goldhammer et al. 2020). In this context, response accuracy, the selected response category, and the estimations of ability based on these have been referred to as the “result data” in this study. The result data, which are commonly used, provide valid and reliable solutions to numerous issues in measurement and evaluation. However, while these data include important information regarding how well the task was completed (Zhang et al. 2023), they have certain limitations in providing insights into the behavioral differences in the manifestation of performance (Goldhammer et al. 2017).

In computer-based assessments, all actions during the interaction with the computer can be automatically captured, and as a result, log files that record the actions with time stamps are generated (Han and Wilson 2022; Jiao et al. 2021). Accordingly, human–human or human–computer interaction data recorded in computer-based assessments, which provide more detailed information not only about how well problems are solved but also about how they are solved and reflect problem-solving processes, are referred to as the “process data” (Fu et al. 2023b; Zhan and Qiao 2022).

Empirical findings have been obtained in some studies, suggesting that including process data in ability estimation has increased the accuracy of ability estimates (Reis Costa et al. 2021), resulted in a better reliability of estimates (Scalise and Clarke-Midura 2018; Zhang et al. 2023), decreased the standard errors of ability estimates (Wang et al. 2019), and led to a better prediction performance of ability estimates based on process data compared to those based solely on item responses (Tang et al. 2020). At the same time, these data also make it possible to understand individuals’ strategies related to the solution (Liu et al. 2018). For example, Teig et al. (2020) expressed that individuals may not perform in the same way during the process, even if they have similar scores. Some individuals may reach the correct response by applying the correct strategy, while others may do so by trial and error or a random approach. Similarly, among individuals who scored low, there may be some who failed by partially applying the correct strategy, while others did not use any correct strategy at all.

When studies related to process data are examined, complex problem-solving (CPS) skills, which consist of simulation tasks and are regarded as one of the important 21st-century skills due to their non-routine nature, draw attention (Greiff et al. 2015b; H. Xu et al. 2018). This skill, with open-access log files, is comprehensively evaluated in the PISA 2012 cycle. Buchner (1995) characterizes CPS as the effective engagement with the ever-changing task environments, environments that evolve due to user actions or over time, where the underlying principles become apparent only through methodical investigation and the assimilation of the information gathered in that process. Buchner’s definition requires the acquisition of knowledge about the problem situation and the application of this acquired knowledge to solve the problem (Herde et al. 2016). These two phases, essential for CPS performance, were defined by Funke (2001). According to Funke, during the knowledge acquisition phase, the participant needs to understand the relationships between variables and the system’s dynamics. In the knowledge application phase, the knowledge acquired in the first phase is used to achieve the goal state specified in the problem. It is a common assumption that there is a causal relationship between the knowledge acquisition phase and the knowledge application phase, with the former being a necessary and sufficient condition for the latter (Funke 2001). In their study on the construct validity of CPS, Greiff et al. (2013a) found a high correlation between the two dimensions. Similarly, Greiff et al. (2013b) also concluded that the construct consists of two dimensions: knowledge acquisition and knowledge application. Wüstenberg et al. (2012) confirmed the two-dimensional structure and stated that knowing the rules of relationships between variables is a prerequisite for applying those rules.

According to Dörner and Funke (2017), measures of CPS skills must not be reduced solely to the result of the solution. When measurement tools require more exploration and actions, richer log files are formed (Greiff et al. 2016). Tasks measuring CPS skills, due to their characteristics, generate comprehensive log files and provide an important source of information for examining the relationship between behaviors during the problem-solving process and overall performance (Stadler et al. 2019; Greiff et al. 2015b).

Research related to CPS and process data encompasses several areas: improving the understanding of the construct (Greiff et al. 2013a, 2013b; Molnár et al. 2013; Sonnleitner et al. 2013; Wüstenberg et al. 2012), investigating the strategies used in solving these problems and exploring the potential of log files (Greiff et al. 2016, 2018, 2015b; Ren et al. 2019; Stadler et al. 2019; Wüstenberg et al. 2014), proposing new statistical models utilizing log files (Chen et al. 2019; Fu et al. 2023a, 2023b; Zhan and Qiao 2022; H. Xu et al. 2018), suggesting methods/approaches for feature extraction from log files (Han et al. 2019), building prediction models in the context of machine learning using features derived from log files (Pejić and Molcer 2021), and applying various clustering methods such as biclustering (X. Xu et al. 2024). Early period research generally focused on understanding the construct, while recent studies have shifted toward new modeling approaches.

In some studies related to CPS, only result data or process data were utilized, while in others, both types of data were utilized. In some studies, result data such as academic grades, item accuracy, and reasoning tests were employed (e.g., Greiff et al. 2013a; Wüstenberg et al. 2012). On the other hand, only process data were utilized in many studies (e.g., Chen et al. 2019; Greiff et al. 2015b, 2016; Pejić and Molcer 2021; Stadler et al. 2019; Ren et al. 2019; Liu et al. 2018; Qiao and Jiao 2018; H. Xu et al. 2018; X. Xu et al. 2024). However, studies in the context of CPS that utilize both process data and result data are relatively limited (e.g., Scherer et al. 2015; Zhan and Qiao 2022; Wüstenberg et al. 2012). In this context, it can be said that more comprehensive and deeper interpretations of individuals’ performance can be made by using both types of data together.

The PISA data utilized in this study serves as an appropriate source for the application of data mining (machine learning) methods due to its provision of large-scale datasets (Yu et al. 2012). Tree-based methods are gaining attention in this field. However, there is an instability problem in single trees, which is one of these methods (Strobl 2013). Due to this instability, the predictions obtained from single trees exhibit high variability (Strobl et al. 2009). In this case, single trees can lead to very different splits even with a small change in the training data (Hastie et al. 2009).

As a solution to this, it is stated that the use of ensemble learning methods can significantly improve the prediction performance of single trees (James et al. 2013). In these methods, a prediction model is created by combining the strengths of an ensemble of simpler models (Hastie et al. 2009; Strobl 2013). On the other hand, the CART algorithm, due to its simultaneous selection of variables and cut-off points, may lead to variable selection bias in favor of independent variables with more possible splitting points (more categorical or continuous) (Hothorn et al. 2006). This bias has been demonstrated for the random forest algorithm by Strobl et al. (2007). At the same time, the lack of statistical significance in the algorithm is also considered as a limitation (Mingers 1987).

To address these issues, the conditional inference tree algorithm, which integrates non-parametric and tree-based regression models with conditional inference theory (Hothorn et al. 2015), was proposed by Hothorn et al. (2006). In conditional inference trees, the stopping rule and significance level of the relationship between dependent and independent variables are determined based on statistical tests (Hothorn et al. 2006). In this study, variables of different scale types have been utilized, and certain variables have a broad range. Therefore, the rarely used gradient boosting algorithm based on conditional inference trees (shortened as conditional gradient boosting) was utilized. In this algorithm, gradient boosting is applied, with conditional inference trees as base learners (Hothorn et al. 2024).

The fundamental principle of gradient boosting is to create a strong learner by combining the results of numerous weak learners (Hastie et al. 2009). The algorithm was developed by Friedman (2001, 2002). In this algorithm, the aim is to improve the prediction by assigning extra weight to the parts that were misclassified in previous stages of the sequentially constructed trees (Liaw and Wiener 2002).

In research on CPS, the combined use of process data and result data has been found to be limited. In addition, studies that address CPS, process data, and machine learning algorithms together are limited. Studies conducted by Qiao and Jiao (2018) and Pejić and Molcer (2021), which can be considered within this scope, utilized methods based on classification trees. Moreover, as far as is known, no study that addresses the conditional gradient boosting algorithm, where regression trees serve as base learners, in an educational dataset containing both process data and result data related to CPS has been found.

Therefore, this study aims to examine the relationships between process data obtained from the log file of the first item of the climate control unit in the PISA 2012 Türkiye dataset, as well as result data encompassing various cognitive and affective constructs, both separately and together with CPS, and to determine the variable importance rankings. With the obtained findings, it is intended to contribute to the literature by providing a deeper interpretation of the construct through the combined consideration of process data and result data related to CPS, supporting the operation of effective formative feedback mechanisms for tests measuring CPS, offering insights into regulations for test development processes, and exemplifying the use of the conditional gradient boosting algorithm in an educational dataset. Accordingly, the research questions are as follows:

RQ_1_: What are the performance levels of predicting CPS skills using the conditional gradient boosting algorithm with process data obtained from the first item of the climate control unit, various result data, and a dataset that combines both types of data?

RQ_2_: What are the variable importance rankings in predicting CPS skills across the three datasets?

## 2. Materials and Methods

The datasets and log files were accessed from https://www.oecd.org/pisa/pisaproducts/database-cbapisa2012.html, (accessed on 15 December 2022). In this study, individuals who answered the first item (CP025Q01) of the climate control unit were included, resulting in an initial sample of 994 participants. During the calculation of the “number of apply” variable (one of the process data variables), individuals who had never clicked the “apply” button were excluded from the analysis because clicking this button is necessary to explore the relationships among the variables in the problem-solving process. Consequently, those who did not perform this action made no meaningful attempt to solve the problem. As a result, analyses were conducted on a final sample of 915 participants.

### 2.1. Creation of the Dependent Variable

The dependent variable, the CPS score, was measured using a computer-based test. For countries that participated in the computer-based problem-solving assessment in PISA 2012, there were eight different forms, ranging from 31 to 38. This assessment measured problem-solving skills using 42 items, of which 15 were static and 27 were interactive (complex) (OECD 2014). Consequently, the problem-solving score reported in PISA 2012 was calculated based on the responses gathered from these two categories of problems. However, because this study focused specifically on CPS skills, the CPS score was computed using a model-based approach, informed by raw scores obtained only from the complex problems.

In the ability estimation process, data from 2022 individuals encompassing all forms that include CPS items (Forms 31 to 38) were used. An estimation under the generalized partial credit model (GPCM) and the partial credit model (PCM) was carried out using the expectation–maximization algorithm (Bock and Aitkin 1981) with a full-information maximum likelihood approach in the TAM package (Robitzsch et al. 2024) in R 4.4.2 (R Core Team 2024). For estimations based on the graded response model (GRM), the ltm package (Rizopoulos 2006) was employed. Student weights were included in all estimations. According to the -2LL, AIC, and BIC criteria, the lowest values were obtained under GPCM.

To conduct estimations using IRT-based models, certain assumptions must be met. Accordingly, to test the unidimensionality assumption, estimates were generated for GPCM, which showed the best fit, for one-, two-, and three-dimensional solutions, and the model–data fit results were examined. The best fit was provided by the one-dimensional model, indicating that the unidimensionality assumption was satisfied. For local independence, Yen’s Q3 test was applied to the one-dimensional model. When no values exceed 0.2 in the residual correlation matrix, local independence is considered satisfied (Chen and Thissen 1997). In this study, those values ranged between −0.22 and 0.13, demonstrating that the local independence assumption was met.

Within the TAM package, ability estimations can be performed using weighted likelihood (WLE), maximum likelihood (MLE), and the default expected a posteriori (EAP) approaches. Among these, the highest reliability emerged under the EAP approach; therefore, the EAP estimates were used as the ability scores. These scores ranged from −2.135 to 2.638. The ability scores were then scaled to have a mean of 500 and a standard deviation of 100. In this study, data from individuals who received Forms 31, 32, 35, and 36, which included the CP2 cluster containing the climate control unit, were utilized for their ability scores.

### 2.2. Climate Control Unit

The first item from the climate control unit was used in the creation of the process data variables. This item is consistent with the theoretical framework of CPS and effectively represents these skills (Greiff et al. 2015b). In this problem, the vary-one-thing-at-a-time (VOTAT) strategy (Tschirgi 1980) must be employed (Herde et al. 2016). The strategy has attracted considerable attention in CPS research, and its usage is directly linked to a better overall CPS performance (Greiff et al. 2018). In the context of the PISA 2012 problem-solving assessment, the use of the VOTAT strategy was found to correlate strongly and positively with both performance on the climate control item and overall problem-solving performance at the individual and country levels (Greiff et al. 2015b). Additionally, this item is highly suitable for feature extraction due to having a comprehensive log file. It has frequently been used as a source for understanding the nature of these skills in various CPS assessments (e.g., Chen et al. 2019; Greiff et al. 2015b; Han et al. 2019; Pejić and Molcer 2021; H. Xu et al. 2018). For these reasons, it was chosen for this study.

There are two approaches for measuring CPS skills: the linear structural equation (LSE) approach and finite state automata (FSA). While LSEs provide a framework for modeling linear relationships among quantitative variables, FSA is used to model relationships among qualitative variables. In the LSE approach, the relationship between the system’s causal structure is explored by utilizing the changes in output variables caused by the manipulated values of input variables during the knowledge acquisition phase. The knowledge acquisition phase in the FSA approach is similar. In the LSE framework, during the knowledge application phase, the goal state specified in the problem must be reached for output variables with the help of input variables. In the case of FSA, the knowledge application phase involves finding a path from the initial state to the goal state (Funke 2001).

With the two approaches defined by Funke (2001), complex problems have been expressed in common frameworks, addressing the issue of comparability in CPS research and making the design of new problem situations more flexible (Greiff et al. 2015a). However, while Funke’s (2001) approach solved the comparability issue, it did not address the scalability problem. In this context, during the early periods of CPS measurement, the use of a single task led to various limitations in the psychometric properties of the test (Greiff et al. 2015a). To address these limitations, multiple complex systems involving several tasks have been proposed. For this purpose, the MicroDYN and MicroFIN approaches have been defined. Accordingly, the MicroDYN approach consists of problems based on LSEs, while the MicroFIN approach involves problems based on FSA (Greiff et al. 2015a).

**Figure 1:**
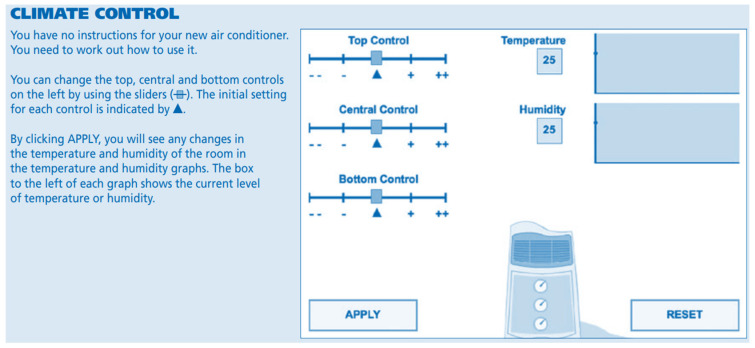
The information screen of the climate control unit (OECD 2014).

The items used in the unit in the study are of the MicroDYN type (OECD 2014) and do not require domain-specific prior knowledge (Greiff et al. 2018). In MicroDYN problems, which represent key aspects of CPS, the main tasks involve discovering and controlling causal relationships in an unknown system (Funke 2010; OECD 2014). In the climate control unit, during the knowledge acquisition phase, individuals need to control up to three input variables (OECD 2014). Here, individuals explore the system freely. For example, at this stage, they may reset the system and begin exploration again (Funke 2010). Subsequently, the knowledge about the discovered rule must be displayed (OECD 2014). This is performed through diagram drawing. This drawing, which can also be referred to as the externalization of the mental model, allows for the evaluation of the acquired causal knowledge (Funke 2010). In the second phase, the knowledge application phase, individuals are required to control the system to reach the target output values by selecting the appropriate input levels (Funke 2010). At this stage, to ensure conceptual independence between the knowledge acquisition and knowledge application dimensions, the correct diagram is displayed on the user’s screen (Greiff et al. 2018). In terms of the unit preferred for this study, the first item is related to the knowledge acquisition phase, and the second item is related to the knowledge application phase (OECD 2014). In accordance with these explanations, a screenshot of the relevant unit is provided in Figure 1.

According to Figure 1, it is stated that the top, central, and bottom control bars on the left (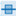) can be used to figure out how the air conditioner works without a user manual. The initial position of each setting is indicated by (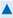). When the “apply” button is clicked, changes in the room’s temperature and humidity levels can be seen on the temperature and humidity graphs. The box on the left of each graph shows the current temperature and humidity levels.

**Figure 2:**
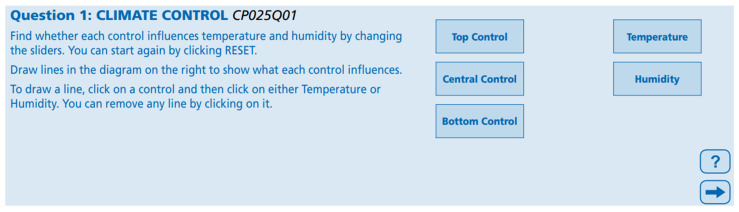
The first question of the climate control unit (OECD 2014).

In the first question used in this study and presented in Figure 2, individuals are asked to slide the control bars left and right to determine whether each bar changes the temperature and humidity values (OECD 2014).

According to the instructions, a retry can be made by clicking the “reset” option. It is requested that the effects of each bar be shown by drawing arrows in the figure. To draw an arrow, one must click on one of the top, central, or bottom control bars and then click on the temperature or humidity boxes. Any arrow can be removed by clicking on it. There are no limitations on the number of trials for exploration in this question. Full points are awarded if the diagram is drawn correctly, while only partial points are given if the diagram is not drawn correctly, with the relationships between variables being explored by changing only one input variable at a time (OECD 2014).

### 2.3. Process Data Variables Created in the Current Study

In studies involving process data analysis, it is frequently observed that the “total time” variable, representing the duration from the start to the completion of the task, is included. Response times in computer-based tests are automatically recorded and serve as a natural data type for investigating cognitive processes (De Boeck and Jeon 2019). Various findings have emerged in research examining the relationship between the overall task duration (Goldhammer et al. 2017) and CPS. For example, Greiff et al. (2016) identified an inverted-U relationship between CPS skills and the time spent on the task. In other words, spending too little or too much time on the task was associated with a lower CPS performance. On the other hand, Scherer et al. (2015) reported a moderate positive relationship between CPS performance and total time. Given the variable’s different types of relationships with performance, the duration variable was included in this study

The other variable obtained is the “VOTAT strategy score”, which is crucial for solving the problem. According to this strategy, when encountering a negative outcome, the variable believed to have caused that outcome is changed, while the other variables are kept constant (Tschirgi 1980). Greiff et al. (2015b) concluded that the VOTAT strategy serves as a valid indicator of broader strategic competencies in problem solving. Various studies have found a positive relationship between this strategy and CPS (Greiff et al. 2016; Stadler et al. 2019). Its importance in explaining CPS performance has also been highlighted in other research (Chen et al. 2019; Pejić and Molcer 2021). In a study by Wüstenberg et al. (2012), the likelihood of constructing a correct model without using the VOTAT strategy, except for the easiest item, was only 3.4% on average. In light of these findings, creating a variable related to the VOTAT strategy was deemed important. For an individual to be considered as having applied this strategy, two input variables (the upper, middle, and lower control bars) must remain at the “0” position while the other variable’s position is changed. Accordingly, partial scoring for this variable was carried out as follows: 0 points if the VOTAT strategy was not used on any of the three input variables, 1 point if it was used on only one variable, 2 points if it was used on two variables, and 3 points if it was used on all three variables.

Various process data can provide insights into the planning and regulation behaviors that individuals exhibit while solving a complex problem. Within this scope, information regarding the “duration of waiting” has been utilized in various studies (Klotzke and Fox 2019; Qiao and Jiao 2018). This variable represents the difference between the moment the task begins and the first action performed by the student. For instance, based on a novel statistical modeling approach, Chen et al. (2019) found that individuals who read the task instructions more quickly and initiated actions sooner in a CPS environment tended to complete the problem with lower accuracy and in a shorter duration. It is also possible to derive various process data variables concerning the frequency of the actions individuals perform. In this context, the “number of actions” variable has been used in log file analyses (e.g., Gobert et al. 2015; Teig et al. 2020). Researchers have examined the relationships between this variable, which represents the total number of actions recorded during a task, and CPS skills. Several studies summarized by Goldhammer et al. (2017) highlight its significance. For example, in a study using PIAAC problem-solving assessment data, Naumann et al. (2014) reported that the number of actions was an important predictor of task success, and there was an inverted-U relationship between action count and task success. The differences among low, medium, and high numbers of actions were associated with factors such as participants’ level of engagement, potential distraction, or confusion about how to proceed with the task. He et al. (2019) reported that action count and total time were the most dominant features when they conducted a clustering analysis using various variables derived from log files. Their findings also indicated that individuals who spent more time and performed more actions generally achieved higher problem-solving scores. Another variable used in this study is the “number of reset” events, reflecting how many times participants reset the system while attempting to solve the problem. The frequency of restarting the task may be related to problem-solving performance. According to Chen et al. (2019), using the reset button increased the probability of solving the problem correctly and also extended the time needed to complete it. In another study that employed hierarchical modeling, at both the process and student levels, the number of reset operations was found to be related to ability estimates at both levels (Liu et al. 2018). Another variable created here is the “number of apply”. In the problem, clicking the “apply” button is necessary for any exploratory actions to be recorded, so the number of times this button is clicked is captured as the “number of interventions”. There is also a variable that reflects clicking “apply” without making any changes to the system, called “observation without intervention”. This refers to at least one instance in which all variables remain at “0” before “apply” is clicked (Greiff et al. 2016). In one study, students who observed without intervention and clicked the “apply” button fewer times performed better in solving CPS tasks (Greiff et al. 2016). In that same study, the scoring process accounted for whether participants performed an observation without intervention at least once. In the present study, the number of times this process was executed ranged between 0 and 33, suggesting that repeatedly performing the same action may relate to participants’ attentional processes (Chen et al. 2019). Therefore, two different variables were created for this situation. The first is a binary “non-intervention observation score” variable scored as 0 or 1, indicating whether this action was performed at least once. The second is the “number of non-intervention observations”, representing how many times participants performed this action.

Furthermore, according to the “number of VOTAT” variable used in the research by Pejić and Molcer (2021), those who experimented with all three control bars equally demonstrated the highest level of success on the task. Based on this, three additional variables that reflect how often the VOTAT strategy was used for each control bar (top, central, and bottom) in solving the problem were created.

An additional data-driven approach was adopted alongside the previously described variables. First, it was noted that the number of VOTAT applications varied among individuals, so each individual’s “total VOTAT applications” were treated as a separate variable. Another variable created was the “duration until first intervention”, defined as the duration from the start of the task to the first click of the “apply” button. This measure was considered potentially informative as an indicator of when exploration begins.

Also included was the “number of times the diagram button” was clicked, as correctly mapping the relationships between variables is required to earn full credit on the task (OECD 2014). Another variable, called the “duration of the last check”, was introduced to capture whether individuals reviewed their work before submitting their responses. It is calculated as the difference between the timestamp of the last action and that of the preceding action. Finally, a speed variable was calculated by dividing the total number of actions by the total time spent on the task.

### 2.4. Result Data Variables Used in the Study

Research shows that there are significant relationships between CPS, reasoning, and academic achievement. Greiff et al. (2013a) found that CPS is moderately related to reasoning skills and predicts academic performance in subjects such as mathematics, physics, chemistry, and biology. Similarly, Wüstenberg et al. (2012) reported a positive relationship between CPS and academic achievement. Sonnleitner et al. (2013) indicated that CPS shows significant positive correlations with reasoning, mathematics, reading literacy, and science performance. Moreover, in the PISA 2012 assessment, high correlations were reported between mathematical literacy, science, reading literacy, and overall problem-solving performance (0.81, 0.78, and 0.75, respectively) (OECD 2014). These findings suggest that CPS may play a key role in enhancing academic success. It has also been stated that a high proficiency in CPS can help individuals effectively tackle problems in various domains (Greiff et al. 2018). Molnár et al. (2013) reported an overall correlation of about 0.35 between domain-specific problem solving and CPS. In addition, the VOTAT strategy, which is crucial for solving the problem used in this study, is considered central to the experimental method in science (OECD 2014). Consequently, this study employed the first plausible values of students’ mathematical literacy, science, and reading literacy scores from the PISA 2012 assessment as the result data.

CPS encompasses not only cognitive skills but also motivational, behavioral, and affective components (Greiff et al. 2018). Building on the statement in the PISA 2012 definition of problem solving, which mentions “…the willingness to engage with such situations in order to achieve one’s potential as a constructive and reflective citizen” (OECD 2014, p. 30), it was considered important to address certain affective traits that may be necessary to solve a complex problem. For instance, Scherer et al. (2015) found significant relationships between CPS and various cognitive and motivational constructs. Similarly, perseverance and openness to problem solving have been reported to be strongly associated with problem-solving performance, particularly at the highest proficiency levels (OECD 2014). Another study examined the relationship between openness to problem solving, perseverance scores, and CPS in data from three different countries included in PISA 2012 (Scherer and Gustafsson 2015). The results revealed positive correlations (ranging from 0.25 to 0.36) between these constructs and CPS, with no substantial differences across countries. Based on these findings, the present study also incorporated students’ scores on openness to problem solving and perseverance, as measured in the PISA 2012 assessment, as part of the result data.

### 2.5. Data Analysis

In this study, missing data were identified for 34% of the participants (312 individuals) in the perseverance variable and again 34% (315 individuals) in the openness-to-problem-solving variable. According to Little’s MCAR test, the missing values were determined to be completely random (*p* = 0.289). Mardia’s multivariate normality test conducted for the perseverance, openness to problem solving, and CPS skills variables indicated that the assumption of multivariate normality was not met. Consequently, parametric techniques were not used, and missing values were imputed using mean substitution, median substitution, and the missForest method. All analyses were repeated for the datasets obtained through these imputation methods. No notable differences emerged in terms of variable importance rankings or metric values; however, histogram plots showed that the missForest approach provided the most suitable results regarding skewness and kurtosis. Therefore, all analyses were carried out using the datasets imputed with the missForest method.

Hyperparameter tuning and model building were performed by incorporating student weights. To assess the suitability of the models, several metrics were employed: mean average error (MAE), mean absolute percentage error (MAPE), bias, mean squared error (MSE), root mean squared error (RMSE), and correlation (R) between actual and predicted values (calculated via the Metrics package; Hamner and Frasco 2018), as well as explained variance (R^2^) (calculated via the caret package; Kuhn 2008). When evaluating model performance, lower MAE, MAPE, MSE, and RMSE values are preferred, alongside bias values close to zero, as well as higher R and R^2^ values. For interpreting the most influential variables, their rankings in terms of variable importance were examined. As noted by Strobl (2013), while the rankings of influential variables may appear similar across different algorithms, their absolute importance scores might differ due to the chosen hyperparameters and dataset characteristics. Hence, the absolute importance scores should neither be compared nor interpreted; only the rankings should be taken into consideration.

A total of 70% of all datasets were used for training, while 30% were used for testing. For the conditional gradient boosting analyses, the mboost package (Hothorn et al. 2024) was used. In the calculations carried out with the blackboost function in this package, the arbitrary loss function was kept at the default Gaussian setting, while the mstop parameter (the number of iterations) and the maxdepth parameter (the maximum tree depth) were fine-tuned using a grid search approach combined with 10-fold cross-validation.^1^

## 3. Results

The metric values obtained for the first research question are given in Table 1.

*RQ_1_:*  *What are the performance levels of predicting CPS skills using the conditional gradient boosting algorithm with process data obtained from the first item of the climate control unit, various result data, and a dataset that combines both types of data?*

Based on Table 1, it can be said that process data demonstrated a moderate predictive performance, result data demonstrated a moderate-to-good predictive performance, and the combined process + result data demonstrated a good predictive performance. Acceptable levels of predictive performance were obtained across all datasets.

*RQ_2_:*  *What are the variable importance rankings in predicting CPS skills across the three datasets?*

**Figure 3:**
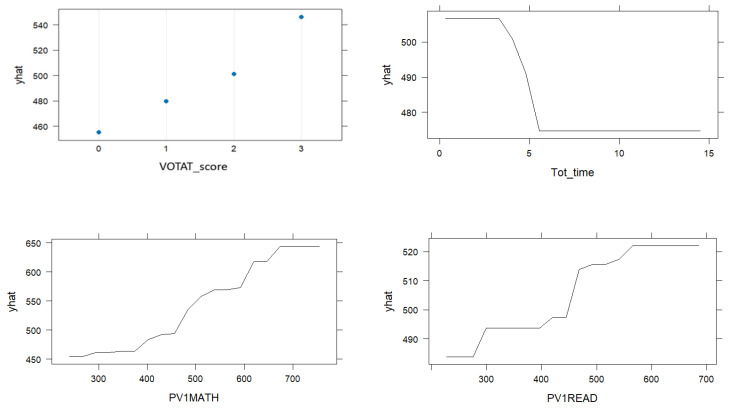

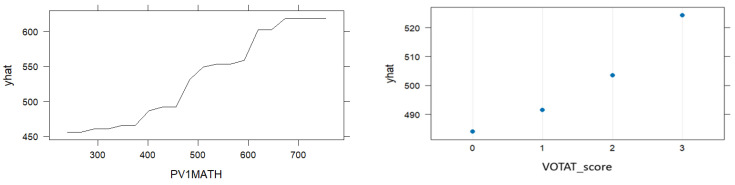
Partial dependence plots. The first row of graphs corresponds to the process data; the second row corresponds to the result data, and the third row corresponds to the process + result data.

For this research question, Table 2 presents the rankings of variable importance values, while Figure 3 shows the partial dependence plots for the top two most influential variables.^2^

According to the variable importance rankings for process data in Table 2, the two most important variables appear to be the VOTAT score and total time. Based on the first row of graphs in Figure 3, as the use of the VOTAT strategy increases, CPS performance also increases. The second most influential variable, total time, shows a negative, nonlinear relationship with CPS performance according to the partial dependence plot. In other words, shorter durations are associated with higher CPS scores. As the time approaches five minutes, CPS scores tend to decline. After five minutes, CPS scores are not significantly affected but remain at relatively low levels.

According to the variable importance rankings for the result data in Table 2, mathematical literacy and reading literacy scores appear to be the two most important variables. Based on the partial dependence plots in the second row of Figure 3, both skills show a strong and positive relationship with CPS.

According to the variable importance rankings for the combined process + result data in Table 2, mathematical literacy and VOTAT scores appear to be the two most important variables. The partial dependence plots in the third row of Figure 3 indicate a positive relationship between these two variables and CPS performance.

## 4. Discussion and Conclusions

The current study aims to examine the predictive performance of process and result data on CPS using a conditional gradient boosting algorithm. According to the findings related to the first research question, process data demonstrated moderate predictive performance, result data showed moderate-to-good performance, and the combination of process data and result data exhibited good predictive performance. All datasets provided acceptable results. Previous studies employing machine learning algorithms in the context of CPS have reported that process data alone yielded good predictive performance (Pejić and Molcer 2021; Qiao and Jiao 2018). However, another study conducted by Zhan and Qiao (2022), examining both process and result data in the CPS context, emphasized that predictions based on process data better reflected an individual’s ability level compared to those relying solely on result data. Furthermore, studies using process data in different contexts have highlighted its contribution to measurement and evaluation practices. Previous findings in the related literature indicate that ability estimation based on process data more accurately reflects an individual’s ability level compared to estimations relying solely on result data, thereby enhancing the precision, reliability, and performance of ability predictions (Reis Costa et al. 2021; Scalise and Clarke-Midura 2018; Tang et al. 2020; Zhang et al. 2023). He et al. (2019) underscored that process data could be particularly informative for identifying additional interventions when individuals respond incorrectly to questions. In addition, Zhang et al. (2023) concluded that incorporating process data into ability estimation allows for reliable predictions even with shorter tests. Consequently, it seems reasonable to state that integrating process data into assessments contributes to test development processes.

Moreover, it is worth noting that the combined use of process data and result data expands the scope of information obtainable about individuals’ abilities, and these data types complement each other. The joint use of these two data types is believed to facilitate a more holistic understanding of the latent trait being measured, thereby increasing the validity of score interpretations. On the other hand, it is crucial to acknowledge that each type of data has its own advantages and limitations. Therefore, when deciding on the type of data to use, it is recommended to consider factors such as the study purpose, the conditions under which the test is administered, and the test format.

Considering the findings related to process data for the second research question, the most important variables, based on variable importance rankings, were the VOTAT score and total time. Numerous studies underscore the significance of VOTAT usage in CPS assessments and its positive relationship with performance (Chen et al. 2019; Greiff et al. 2015b, 2016, 2018; Han et al. 2019; Stadler et al. 2019; Teig et al. 2020; Wüstenberg et al. 2012). This study also concluded that increased use of this strategy was associated with improved CPS performance. Meanwhile, the relationship between total time and CPS performance was found to be negative and nonlinear. Accordingly, shorter completion times were associated with higher CPS scores. Previous research findings on the relationship between time and CPS performance vary. For instance, Scherer et al. (2015) found a moderate, positive relationship between the two variables. Conversely, Csányi and Molnár (2023) reported no significant relationship. Greiff et al. (2016), however, identified an inverted U-shaped relationship between time and the two dimensions of CPS performance. Therefore, it is essential to carefully interpret the results of this nonlinear relationship. Spending too little time may indicate insufficient effort, whereas spending excessive time could signal low proficiency or technical difficulties (OECD 2024). In conclusion, incorporating strategy scores and time as variables in ability estimation is likely to yield more reliable results, contributing to a better understanding of differences in student performance.

According to the variable importance rankings of the result data within the scope of the second research question, the most important variables were mathematical literacy and reading literacy scores. The study results showed a positive relationship between these two skills and CPS performance. Similar findings regarding the relationship between CPS performance and mathematics or reading literacy have been reported in previous studies (Greiff et al. 2013a; Molnár et al. 2013; Sonnleitner et al. 2013). Furthermore, various studies have indicated that CPS performance predicts school grades and is associated with general cognitive ability (Greiff et al. 2013b; Scherer et al. 2015; Wüstenberg et al. 2012). In light of these findings, it is plausible to conclude that incorporating CPS skill instruction into school curricula could contribute to individuals’ cognitive and academic development.

Regarding the variable importance rankings of the combined process data and result data within the second research question, the most important variables identified were mathematical literacy and VOTAT scores. Both variables demonstrated a positive relationship with CPS performance. The dominant effect of mathematical literacy scores in the variable importance graph likely has specific underlying reasons. For instance, Greiff et al. (2018) suggested that high proficiency in CPS would facilitate solving problems across different domains. Similarly, Molnár et al. (2013) found a correlation of 0.35 between CPS and domain-specific problem solving, particularly for mathematics-related problems. The questions in this study correspond to the knowledge acquisition dimension of CPS (OECD 2014). Tasks related to knowledge acquisition within the PISA framework include processes such as “exploring and understanding” and “representing and formulating” (OECD 2014). These processes align with the first two stages of general problem solving defined in PISA and correspond to the problem-solving stages conceptualized by Polya (1945): understanding the problem, devising a plan, carrying out the plan, and looking back. An examination of mathematics textbooks used in schools in Türkiye reveals that the problem-solving sections follow these four stages. Therefore, problem-solving processes are already an integral part of the mathematics curriculum in Türkiye. Although the dynamic structure of the problems used in this study differs from the traditional problems encountered in mathematics classes, they still involve the same problem-solving processes. This similarity may explain the pronounced relationship between mathematics performance and CPS performance.

When all findings are considered together, the current study is thought to shed light on certain issues:

Each data type has unique advantages and limitations. Researchers should select the appropriate data type based on the objectives of the study and the conditions under which the test is administered. Nevertheless, using process data facilitates the mapping of cognitive processes, providing in-depth information about individuals. This flexibility should be factored into test development processes. For instance, the strategy and time variables identified as effective in the study could be incorporated into the test score calculation. Alternatively, test systems that offer warnings and/or feedback during the test session can be created using insights gained from these variables. Because such assessment tools require computer-based implementation, certain limitations may arise, such as accessibility concerns or insufficient technical infrastructure. Consequently, these tools may not be directly applicable to high-stakes exams. However, if they can be deployed on nationwide educational platforms, many of these limitations may be substantially mitigated, and additional instructions for students can be more specifically targeted based on data gleaned from process assessments. For example, a test system that directs students to instructional videos on topics where further support is required, based on information from process data, would save both time and resources. In this way, information from the assessment process could be directly used for instructional purposes. Moreover, the use of process data is not confined to CPS. Such data can also be utilized at various educational levels and in multiple domains, including science (e.g., Scalise and Clarke-Midura 2018; Teig et al. 2020), writing skills (e.g., Deane 2014; Zhang and Deane 2015), and the evaluation of adult competencies in arithmetic, problem-solving, and reading skills (e.g., Goldhammer et al. 2014; He et al. 2019; Klotzke and Fox 2019; Liao et al. 2019; Tang et al. 2020; Zhang et al. 2023), as well as across diverse item types (e.g., Arieli-Attali et al. 2019; Han and Wilson 2022).

It is noteworthy to find out a relationship between CPS, an important 21st-century skill, and success in mathematical literacy, reading literacy, and science. At the same time, it is known that this skill requires multi-step decision-making, as in real-life problems (Funke 2001). In this context, it would be beneficial to incorporate the problem-solving strategies necessary for CPS into school curricula.

The use of conditional gradient boosting algorithms is thought to be an important alternative in complex datasets, such as in this study, which includes related predictors measured on different scales and does not meet the assumptions of parametric techniques.

## 5. Limitations and Suggestions

### 5.1. Practical Limitations and/or Suggestions

Attention should be paid to using the same set.seed value for running necessary functions, as machine learning algorithms involve random processes.

In cases where a dataset carries the risk of variable selection bias due to different scale types, if a method based on conditional inference trees is not employed as is the case in this study, variable transformations may be considered to ensure that all variables fall within similar value ranges.

### 5.2. Suggestions for Future Research

Real-time process data were used in this study. In addition to these data, studies could be conducted using variables that include sequential event patterns (n-gram approach).

This study made use of a log file from a single CPS item requiring the use of the VOTAT strategy. Additionally, only datasets from Türkiye have been used. Therefore, it should not be forgotten that the findings have been interpreted within the context of the conditions examined. Future research may utilize datasets from multiple items requiring different strategies from the PISA 2012 problem-solving assessment and/or data from multiple countries.

Computer-based test systems that include scoring systems incorporating process data into ability estimation, provide real-time feedback to individuals based on process data obtained during the test session, and measure social-emotional skills could be developed.

The dataset used in this study is of moderate size. Research could be conducted to investigate the relationship between the findings and sample size, if any.

## Figures and Tables

**Table 1:** Metric values obtained from the conditional gradient boosting algorithm.

	MAE	MAPE	BIAS	MSE	RMSE	R	R^2^
Process Data	50.369	0.103	1.250	3808.385	61.712	0.742	0.551
Result Data	45.845	0.095	−0.614	3076.156	55.463	0.774	0.599
Process + Result Data	39.501	0.082	−0.758	2278.564	47.734	0.840	0.705

**Table 2:** Variable importance rankings.

	Process Data	Result Data	Process + Result Data
VOTAT score	**1.**	-	**2**.
Total time	**2.**	-	6.
Number of reset	3.	-	4.
Non-intervention observation score	4.	-	7.
Number of bottom VOTAT	5.	-	15.
Number of apply	6.	-	16.
Speed	7.	-	10.
Number of top VOTAT	8.	-	9.
Number of non-intervention observations	9.	-	13.
Number of diagram	10.	-	14.
Number of central VOTAT	11.	-	8.
Number of actions	12.	-	17.
Number of VOTAT	13.	-	18.
Duration of waiting	14.	-	19.
Duration of the last check	15.	-	20.
Duration until first intervention	16.	-	21.
PV1MATH	-	**1.**	**1.**
PV1READ	-	**2.**	3.
PV1SCIE	-	3.	5.
PERSEV	-	4.	11.
OPENPS	-	5.	12.

## Data Availability

The original contributions presented in this study are included in the Supplementary Materials. Further inquiries can be directed to the corresponding author(s).

## Notes

1 The variable importance plots have been provided in the Supplementary Materials Section.

2 The codes related to the analyses have been provided in the Supplementary Materials Section.

## Supplementary Materials

The following supporting information can be downloaded at https://www.mdpi.com/article/10.3390/jintelligence13030029/s1.
