# Supplementary material for: Investigating the Predictive Performance of Process Data and Result Data in Complex Problem Solving Using the Conditional Gradient Boosting Algorithm
Source: J Intell. 2025 Feb 28;13(3):29. doi: 10.3390/jintelligence13030029 (PMC11942880; doi:10.3390/jintelligence13030029)

Supplementary Materials

```
#This script includes calculations specifically for process data.

#The same codes can be applied to other data sets by modifying the data set-related arguments.

data<-readRDS("data.RDS")

#Splitting the dataset into 70%-30%.

n1 <- nrow(data[, c(1:17,23)])

set.seed(123)

train_indices_proc <- sample(1:n1, size = floor(0.7 * n1))

train_proc_data <- data[, c(1:17,23)] [train_indices_proc, ]

test_proc_data <- data[, c(1:17,23)] [-train_indices_proc, ]

############Analyses for Conditional Gradient Boosting################

#Function description

tune_and_train_blackboost <- function(data, dependent_var, weight_col_name = "W_FSTUWT",

                                   mstop_range = (1:10) * 25, maxdepth_range = 1:10,

                                   cv_folds = 10, seed = 123, test_data = NULL) {

  # Installation of the necessary packages

  library(caret)

  library(mboost)

  library(partykit)

  library(Metrics)



  set.seed(seed)


  # Checking if the weight column exists

  if (!(weight_col_name %in% names(data))) {

    stop(paste("The weight column", weight_col_name, "is not found in the dataset."))

  }


  # Seperating and removing the weight column from the data
```

```
weight_col <- data[[weight_col_name]]

data_no_weights <- data[, !(names(data) %in% weight_col_name)]


# Preparation of the formula

formula <- as.formula(paste(dependent_var, "~ ."))


# Defining the tuning grid

tune_grid <- expand.grid(mstop = mstop_range, maxdepth = maxdepth_range)


# Defining train control

train_control <- trainControl(method = "cv", number = cv_folds)


# Model tuning using caret

tuned_model <- train(formula, data = data_no_weights,

                  method = "blackboost", weights = weight_col,

                  tuneGrid = tune_grid, trControl = train_control)


# Extraction of the best parameters

best_mstop <- tuned_model$bestTune$mstop

best_maxdepth <- tuned_model$bestTune$maxdepth


# Training the final model with the best parameters, including the weights

final_model <- blackboost(formula, data = data_no_weights,

                      control = boost_control(mstop = best_mstop),

                      tree_controls = partykit::ctree_control(maxdepth = best_maxdepth),

                      weights = weight_col)


# Return the tuned model, final model, and best parameters

model_results <- list(

  tuned_model = tuned_model,

  final_model = final_model,
```

```
  best_mstop = best_mstop,
  best_maxdepth = best_maxdepth
)

# Evaluation of modeinl performance, if test data is provided
if (!is.null(test_data)) {

  test_data_no_weights <- test_data[, !(names(test_data) %in% weight_col_name)]

  # Making predictions on the test data
  predictions <- predict(final_model, newdata = test_data_no_weights)
  actuals <- test_data[[dependent_var]]

  # Metric evaluation
  mse_value <- mse(actuals, predictions)
  rmse_value <- sqrt(mse_value)
  bias_value <- bias(actuals, predictions)
  mae_value <- mae(actuals, predictions)
  mape_value <- mape(actuals, predictions)
  r_value <- cor(predictions, actuals)
  explained_variance <- caret::postResample(pred = predictions, obs = actuals)[[2]]

  # Adding metrics to results
  model_results$metrics <- list(
    mse = mse_value,
    rmse = rmse_value,
    bias = bias_value,
    mae = mae_value,
    mape = mape_value,
    r = r_value,
    explained_variance = explained_variance
```

```
    )
  }

  return(model_results)
}

# Example usage for process data
results_proc_data <- tune_and_train_blackboost(data = train_proc_data,
                                          dependent_var = "CPS",
                                          weight_col_name = "W_FSTUWT",
                                          test_data = test_proc_data)



# Access final model, tuned model, and metrics
final_model_proc_data <- results_proc_data$final_model
results_proc_data_best_tuned <- results_proc_data$tuned_model[["bestTune"]]
results_proc_data_metrics <- results_proc_data$metrics

# Function for variable importance graph
create_explainer_and_plot <- function(model, data,
                                      target_variable,
                                      weight_variable = NULL,
                                      loss_function = loss_root_mean_square,
                                      seed = 123) {
  library(DALEX)

  set.seed(seed)

  # Exclusion of target and weight variables from data
  data_excluded <- data[, !(names(data) %in% c(target_variable, weight_variable))]
```

```
  # Creation of the DALEX explainer
  explainer <- explain.default(model = model,
                               data = data_excluded,
                               y = data[[target_variable]],
                               weights = data[[weight_variable]])

  # Calculation of variable importance using permutation
  var_importance <- variable_importance(explainer, loss_function)

  # Plotting the variable importance
  plot(var_importance)
}

# Example usage for process data
create_explainer_and_plot(final_model_proc_data,
                          train_proc_data,
                          target_variable = "CPS",
                          weight_variable = "W_FSTUWT")



# Example of a partial dependency graph for a VOTAT_score variable
library(pdp)
final_model_pdp_plot <- partial(final_model_proc_data,
                                pred.var = "VOTAT_score",
                                grid.resolution = 20,
                                train = train_proc_data,
                                type = "regression",
                                plot = TRUE)
print(final_model_pdp_plot)
```

Variable Importance Plots

Figure S1. Variable Importance Plot for Process Data

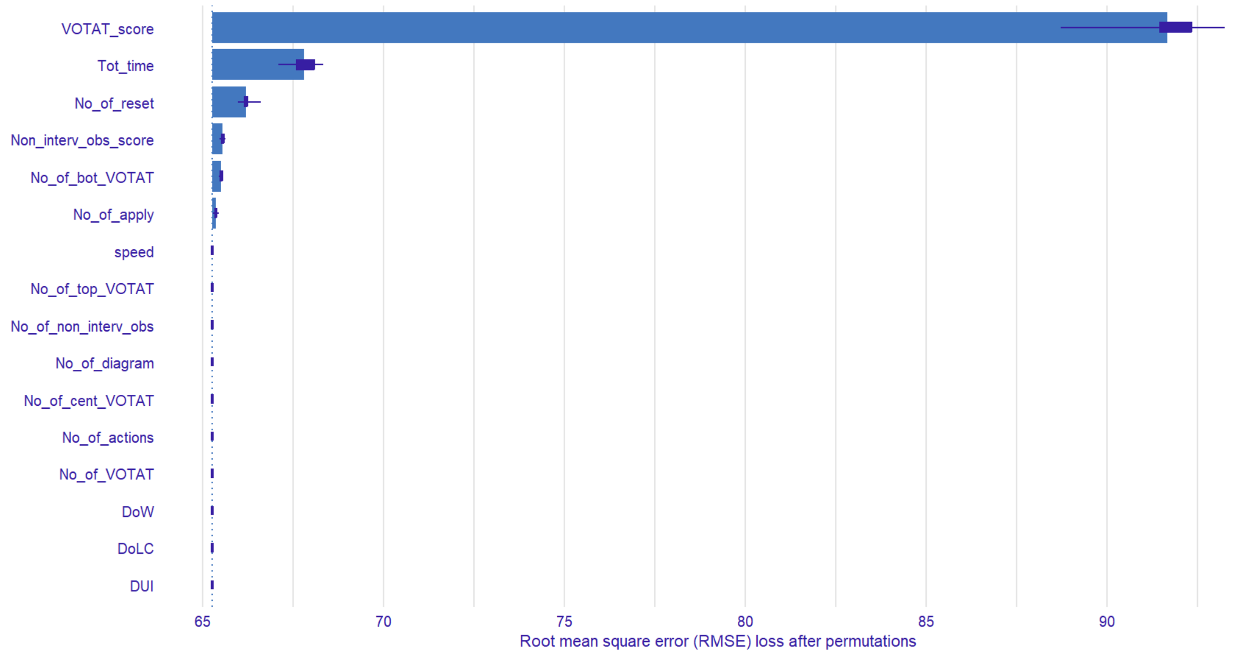

Figure S2. Variable Importance Plot for Result Data

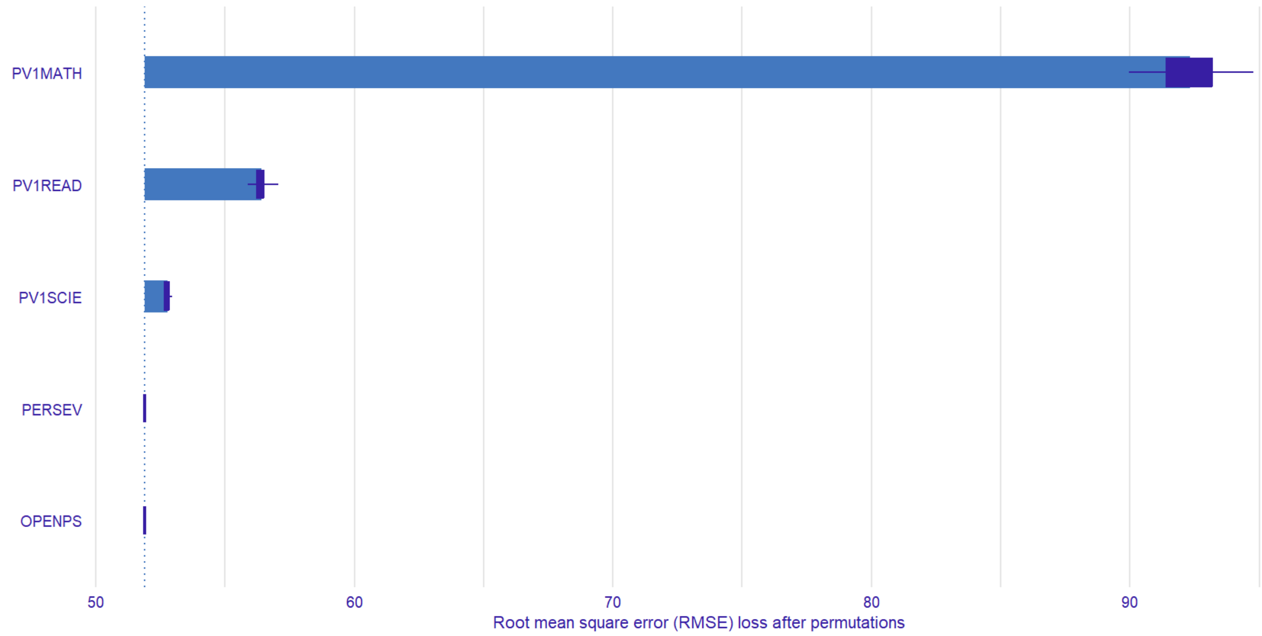

Figure S3. Variable Importance Plot for Process+Result Data

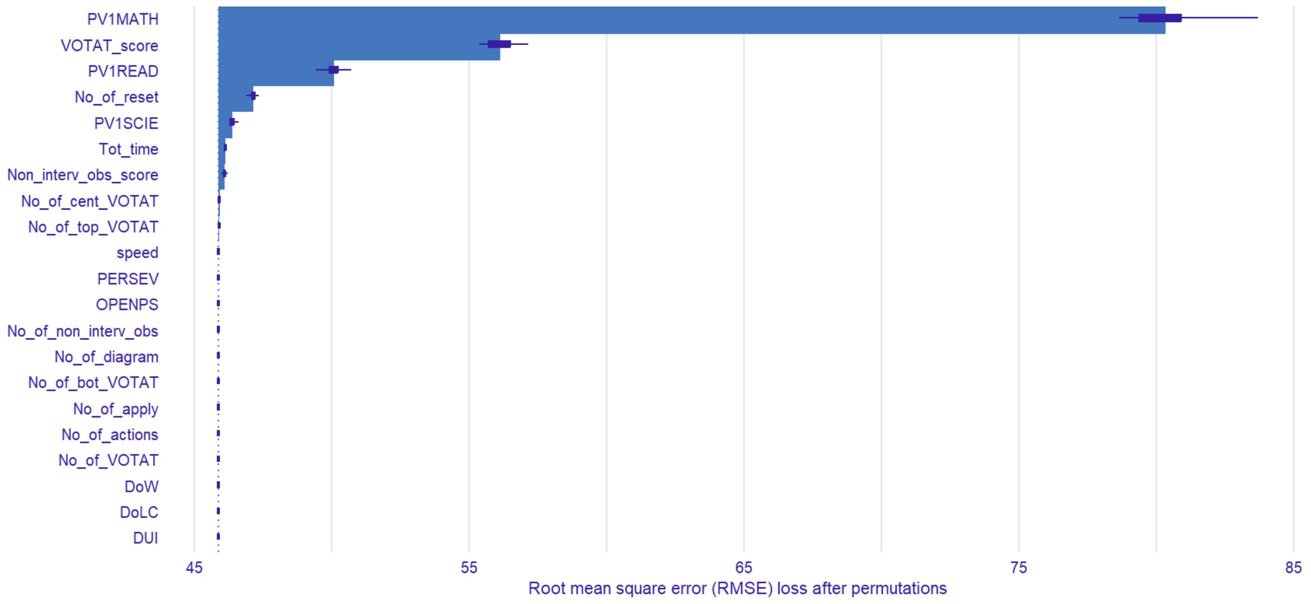

Supplement: Supplementary file 1 [file jintelligence-13-00029-s001.zip › jintelligence-3469859-supplementary.pdf]
